# Low toxicity of dissolved silver from silver-coated titanium dental implants to human primary osteoblast cells

**DOI:** 10.1016/j.toxrep.2024.101776

**Published:** 2024-10-18

**Authors:** Ranj Nadhim Salaie, Alexandros Besinis, Christopher Tredwin, Richard D. Handy

**Affiliations:** a Oral and Maxillofacial Surgery Department, Faculty of Dentistry, Tishk International University, Iraq; b School of Engineering, Faculty of Science and Engineering, University of Plymouth, UK; c School of Dentistry, Faculty of Medicine and Dentistry, Queen Mary University of London, UK; d School of Biological and Marine Sciences, Faculty of Science and Engineering, University of Plymouth, UK

**Keywords:** Nanomaterial, Safety, Metal toxicity, Medical implants, Biocompatibility

## Abstract

the controlled release of silver as a biocide from Ag-coated medical implants is desirable. However, the biocompatibility of Ag leachates is poorly understood. This study investigated the toxicity of silver released from the silver plated titanium implants to human primary osteoblast cells; and the effect of cell culture medium on the silver speciation and bioavailability. Methods: Ti6Al4V discs were coated with Ag nanoparticles (NPs), silver plus hydroxyapatite (HA) nanoparticles (Ag+nHA), or Ag NPs plus microparticles (Ag+mHA). Primary human osteoblast cells were exposed to the leachates from the various discs for up to 7 days. Results: the total Ag concentrations released as leachate from the silver-plated titanium discs were 0.7–1.6 mg L^−1^, regardless of treatment. Cumulative silver release over 7 days was approximately 3 mg L^−1^ in all treatments. The concentration of total Ag in the cell homogenates from all the Ag-containing treatments was modest, ∼ 0.1 µg mg protein^−1^ or less at day 7. Cells showed normal healthy morphology with no appreciable leak of LDH or ALP activity into the external media compared to the reference control. Similarly, there was no significant differences (Kruskal Wallis, *p* > 0.05) in the LDH or ALP activity in the cell homogenate between treatments. Conclusions: overall, there was a controlled release of Ag into the external media, but this remained biocompatible with no deleterious effects on the osteoblast cells, which means that the released silver to the peri-implant environment is not toxic making the coating potential for clinical use

## 1 Introduction

The use of titanium alloy implants in dentistry [1], [2], and in bone surgery [3], [4], is now well established. However, medical grade titanium alloy alone has no inherent biocidal properties to prevent infection, and the metal alloy surface does not especially promote osseointegration [5], [6]. Consequently, there has been interest in developing composite coatings on the titanium alloy that are both anti-bacterial and biocompatible with bone cells. Silver is well-known for its biocidal properties [7], [8], and one such approach is to coat the titanium alloy with nano silver by electroplating, and with a topcoat of nano hydroxyapatite (nHA) to promote osseointegration. Our previous research has shown that such titanium alloy composites are both biocidal to microbes [9], [10] and are also biocompatible with the growth of osteoblast cells [11].

The nano-enhanced surface shows a slow release of dissolved Ag that goes through the porous topcoat of nHA and into the surrounding media [9], and is likely partly responsible for killing the microbes via free metal ion toxicity [7], [8], [12], [13]. However, while this might be a beneficial controlled release of dissolved Ag to provide infection control immediately after surgery. There is also a safety concern for the patient, because dissolved silver can also be toxic to mammalian cells. The target organs for dissolved silver toxicity include vital organs such as the liver and kidney in rodents and humans. In rats, the lethal dose (LD_50_) is around 400 mg kg^−1^ body weight of silver nitrate [14]. In humans, there is no agreed lethal concentration value of silver, but the lethal toxic dose rate of silver is estimated to be higher than 0.5 µg/kg of body weight/day [13]. So, the clinical dilemma is to provide the slow Ag dissolution that imparts antibacterial properties in the wound post-surgery, without presenting an Ag toxicity risk to the wound healing, or the patient.

This problem requires some understanding of the behaviour of any apparent dissolved Ag released by dissolution, and its subsequent behaviour in complex media such as blood, or cell culture media in the case of *in vitro* investigations [15]. The toxicity of dissolved silver in such media is likely dependent on the exposure concentration and time (i.e., the total dose), the chemistry of the cell culture medium and how it alters silver speciation and/or bioavailability, the type of cells used, and the cell density in the culture system (i.e., the dose delivered per cell). In blood, physiological saline or culture medium where typically ∼100 millimoles per litre concentrations of Cl^-^ ions are present, the Ag rapidly forms poorly soluble silver chloride complexes at neutral pH values, which are less bioavailable [16], [17], [18]. The Ag^+^ ion also avidly binds to –SH groups on amino acids and proteins [19], [20], and with high mg L^−1^ amounts of proteins in culture media or blood (e.g., albumin), the often small fraction of Ag^+^ predicted by speciation calculations would, in theory, be rapidly chelated.

Nonetheless, dissolved silver salts can be toxic to cells in culture media, albeit at mg L^−1^ concentrations, decreasing cell proliferation and viability [21]. For example, for silver nitrate, 5 mg L^−1^ AgNO_3_ in cell culture media (DMEM + 10 %FBS + 1 % antibiotic) was found to induce significantly more LDH leak to the external media compared to unexposed controls in human fibroblast cells after 24 hours [22]. The mechanisms of cellular toxicity for dissolved Ag^+^ includes inhibition of the ubiquitous Na^+^ K^+^-ATPase [23] and thus interfere with Na homeostasis. Dissolved silver, and silver nanoparticles, can also cause oxidative stress via damaged proteins or enzymes in the cell [24], [25]. Our previous studies on nano-Ag enhanced titanium alloy implants have identified the optimum amount of Ag for biocidal properties [9], [22], whilst enabling the bone cells to survive [11], the antibacterial dose of Ag was 0.2 mg L^−1^. But with the cells *in situ* and growing on the surface of the implant, it is not possible to conclusively verified the hazard of the dissolved Ag fraction released by dissolution compared to effects of the implant coating itself.

In our previous study (Salaie et al., 2020), it was found that Ag+nHA was biocompatible with human primary osteoblast cells but a slight decrease in cell survival was noticed in Ag alone and Ag+mHA). However, in this study, the aim was to investigate the toxicity of silver released from the silver-plated titanium implants to human primary osteoblast cells to find out whether the decreased cell viability was caused by contact toxicity or ion toxicity; and also to investigate the effect of the cell culture medium on bioavailability of dissolved silver. The specific objectives were to expose the silver coated specimens (without cells present) to the cell culture medium (DMEM + 10FBS + 1 % antimicrobial) so as to condition the media with a ‘dissolved’ silver leachate from silver plated titanium discs; and then transfer the conditioned media to the growing cells in culture plates. A second objective was to measure the dissolved silver uptake by the cell monolayer compared to the total silver release to the external media. Then finally, to study the toxic effect of dissolved silver on the cells by measuring the LDH and ALP activities in the media and cell homogenates, along with electrolytes, and the cell protein content as well as examining the cell morphology *in situ* under the microscope after exposure.

## 2 Materials and methods

### 2.1 Specimen preparation

The procedure for specimen preparation and characterisation was the same as previously described [9], [26]. Briefly, medical grade-five titanium alloy discs (Ti6Al4V) measuring 15 mm in diameter and 1–1.5 mm thickness were polished down to a mirror finish and then silver electroplated in a silver nitrate bath to produce an even layer of silver nanoparticles on top of the surface. Electroplating was done by attaching the silver source to the anode and the titanium disc to the cathode of the electric current (1 voltage for 3 minutes). Both nano and micro HA particles were added to the silver-plated titanium discs by sintering method. To ensure biocompatibility, the discs were further coated with nano or micron-sized hydroxyapatite particles and cured in a furnace. To confirm coating quality and integrity, the specimens were examined by scanning electron microscope (SEM, JEOL/JSM-7001F), with an Oxford Instruments INCA X-ray analysis system attached. The latter with a detecting energy of 15 keV at a working distance of 10 mm. Energy dispersive spectroscopy (EDS), (EDS, spot size, 10 μm; accelerating voltage, 15 kV; working distance, 10 mm) was used to determine the surface composition of the specimens as previously described.

### 2.2 Cell culture

The experiment was performed using primary human osteoblast cells (Hob) obtained from ECACC (European Collection of Cell cultures). Cells were cultured in 75 cm^2^ flasks (Sterilin, Newport, UK) with vented caps, and containing 15 mL of DMEM (Dulbecco’s Modified Eagle’s medium) with L-glutamine, 10 % foetal bovine serum (FBS), and 1 % penicillin-streptomycin (100 IU penicillin-100 μg/mL streptomycin) purchased from Invitrogen. The media were changed as required for the health of the cells (every 3–4 days) and cells were sub-cultured into new flasks when the confluence reached 80–85 %. For the latter, the media was removed first and the cells were washed twice with phosphate buffer saline, D-PBS, (Fischer Scientific, without added calcium and magnesium), then trypsinised with 2 mL of 0.25 % trypsin and EDTA. Cells were re-suspended in fresh culture medium, then counted with a haemocytometer; trypan blue dye exclusion was used to check the cell viability. The cells were maintained at 37 °C in an incubator with a humidified atmosphere of 5 % CO_2_ and 95 % air. Cells at passage 7 and 8 were used for the experiment.

### 2.3 Experimental design

The experimental design was based on our previous study with osteoblasts [11], except that the cells were exposed to media (leachates) from the various discs, rather than grown on the discs directly. The control for the experiment was untreated cells in normal culture media (hereafter termed the ‘reference control’). The treatments were cells exposed to media obtained from silver plated titanium discs (Ag), silver plated titanium disc plus nano HA (Ag+nHA), or silver plated titanium discs plus micro HA (Ag+mHA) respectively. All the discs, as prepared for the treatments above, were sterilised by gamma radiation (radiation dose 36.42–40.72 kGy for 10 hours) prior to the experiment. The discs (without any cells) were initially treated with cell culture media by covering with 1 mL of cell culture medium (DMEM, supplemented with 10 % FBS and 1 % antimicrobials) and incubated in 5 % CO_2_ with 95 % air at 37 °C (HETO-HOLTEN cell house 170) for 2 days, then the media was discarded and the discs were ready for the experiment. This precautionary step enabled the removal of any rapidly exchangeable excess silver from the coatings that might damage the cells [see [11] on the stability of the coatings].

**Fig. 1:**
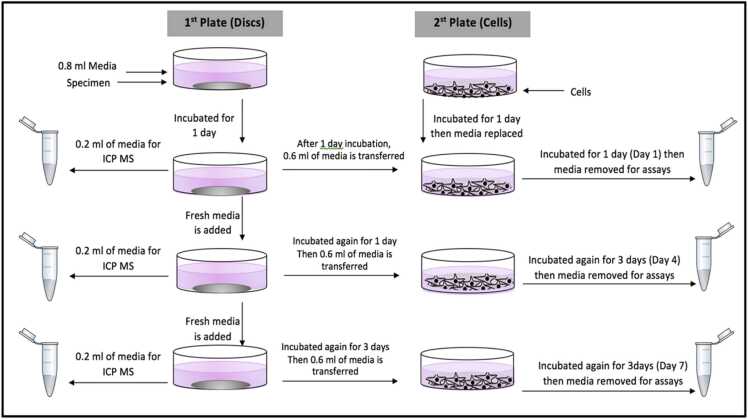
The experimental design, the approach to the experiment involved seeding the cells in one culture plate and treating the samples with the cell free culture medium in another plate. Note the time points on which the conditioned media was exposed to the cells.

The experiment was conducted in 24-well microplates (*n* = 6 plates/treatment). The microplate was the unit of replicate in the study design and the experiment was run on two separate occasions with different batches of the cells, and each run was triplicated (x3 plates/treatment/run). The cells were grown directly on cell culture dishes and in parallel, the discs were incubated with culture media without the cells for the experiment. Briefly, while the discs were incubating in the cell culture medium, at the same time, cells were grown in a second series of plates (*n* = 6 plates/treatment, seeded with 30,000 cells/well). To initiate exposure of the cells, the now conditioned media from the incubation of the discs was added to the cell cultures. The media for the cells were replaced by the conditioned medium from the discs on days 1, 4 and 7 (Fig. 1). Samples of the conditioned media were collected before adding it to the cells (termed ‘before exposure’), and then after exposing the cells to it for fixed durations (termed ‘after exposure’). The silver concentration in the conditioned media was measure before and after the exposure using inductively coupled plasma mass spectrometry (ICP-MS). The media (after exposure) was also analysed for Na^+^, Ca^2+^ and K^+^ using inductively coupled plasma optical emission spectroscopy (ICP-OES), as well as for lactate dehydrogenase (LDH) and alkaline phosphatase (ALP) activities (see below). The media was sampled from cell growing plates at days, 1, 4 and 7.

At the end of the experiment, the cell were photographed *in situ* under the light microscope (Olympus CK30-F200 with a Galaxy s6 camera) to examine the cell morphology and confluence of the dishes. Then, the cells were washed with 2 mL of washing buffer (300 mmol L^−1^ sucrose, 0.1 mmol L^−1^ EDTA, 20 mmol L^−1^ HEPES buffered to pH 7.4 with few drops of Trizma base) and then 1 mL of a lysis buffer [the same as the washing buffer above, except hypotonic with a sucrose concentration of 30 mmol L^−1^, and containing 0.001 % of Triton-X 100 (Sigma Aldrich)] to lyse the cells. The resulting cell homogenate was analysed for total protein content, and LDH and ALP activities. The concentration of total Ag, Na^+^, Ca^2+^ and K^+^ were also measured by ICP-MS or ICP-OES as appropriate. The conditioned media from implants was tested for interference with the endpoints (tests) and no interference with the testes was found.

### 2.4 Biochemistry

Alkaline phosphatase activity is a functional biomarker of osteoblast activity and is essential to the mineralisation of bone. The ALP activity was measured in the external culture media on each day of the experiment and also in the cell homogenates at the end of the experiment (7 days) as previously described [11]. Briefly, for the cell homogenates, 30 μl of sample were added to the well of a 96-well micro plates, with 105 μL of 100 mmol L^−1^ glycine buffer (pH 10), then 145 μL of 500 μmol L^−1^ para-nitrophenylphosphate (pNPP, Acros, UK) was added to each well to start the reaction. The plate was shaken twice and read immediately (VersaMax plate reader, Molecular Devices, Berkshire, UK) for 300 seconds at 405 nm at room temperature, with appropriate sample blanks.

Lactate dehydrogenase (LDH) activity is a well-established biomarker of membrane integrity and was measured every day in the external media to assess any leak of the enzyme from the cells, and in cell homogenates at after 7 days as previously described [11]. Briefly, 100 μL of sample added to the reacting mixture (2800 μL of 6 mmol L^−1^ sodium pyruvate in 50 mmol L^−1^ phosphate buffer at pH 7.4, plus 100 μL of 6 mmol L^−1^ NADH solution), mixed directly in a 3 mL cuvette and the change in absorbance was measured over 2 minutes at 340 nm (Jenway 7315 spectrophotometer). LDH activity was expressed as IU mg L^−1^ (μmol min^−1^ mL^−1^) for the media and μmol min^−1^ mg^−1^ cell protein for the cell homogenate. The ALP activity was similarly expressed. To normalise for cell protein, the protein content of homogenates (10 µL) was measured using the bicinchoninic acid (BCA) method (MC155208, Pierce, Rockford, USA) at 592 nm against bovine serum albumin standards.

### 2.5 Metal and electrolyte analysis

Metal analysis was conducted as previously described [11]. The total silver concentration in the external media and the cell homogenate was measured by ICP-MS (X series model, Thermo Fisher Scientific, UK). The Na^+^, K^+^ and Ca^2+^ concentrations in the external media and the cell homogenate was determined using ICP-OES (iCAP 7400 Radial, Thermo Fisher Scientific, UK). For metal and electrolyte analysis, 400 μL of the external media from each sample were acidified with 20 μL of 70 % nitric acid. For the cell homogenate, 800 μL of sample was mixed with 1 mL of 70 % nitric acid and left overnight to complete the digestion. Samples were analysed against matrix matched standards and with blanks in the sample run to correct for instrument drift, as appropriate. Note the total Ag concentrations are reported in mg L^−1^ and the electrolytes in mmol L^−1^ to enable comparison with our studies.

### 2.6 Statistics

Statgraphics version 16 was used for the statistical analysis. After checking for outliers, skewness and kurtosis, data that were normally distributed were subjected to one way analysis of variance (ANOVA) for treatment or time-effects within treatment, followed by Tukey’s multiple range test to determine the locations of any significant differences. For non-parametric data, the Kruskal Wallis test was used and significant differences were found using Box and Whisker plot. However, for multifactor analysis (e.g., treatment x time), a two way ANOVA was used where appropriate. The default 95 % confidence interval was used for all statistical analysis. All data are expressed as mean ± S.E.M.

## 3 Results

### 3.1 Characterisation of the Titanium alloy Discs

This was done to confirm our routine preparation method was working as expected. Specimens were successfully coated with silver and HA nano or micro particles. SEM and EDS images of Ag showed a continuous layer of silver nanoparticles on titanium. Ag+nHA showed a uniform layer nano HA coating on silver plated titanium, with a few surface cracks as expected. Moreover, the Ag+mHA discs were found to have HA micro particles on the surface with noticeable gaps between the particles to enable the media to access the underlying coat of silver (See Figures S1 and S2).

### 3.2 Silver release to the external media

**Fig. 2:**
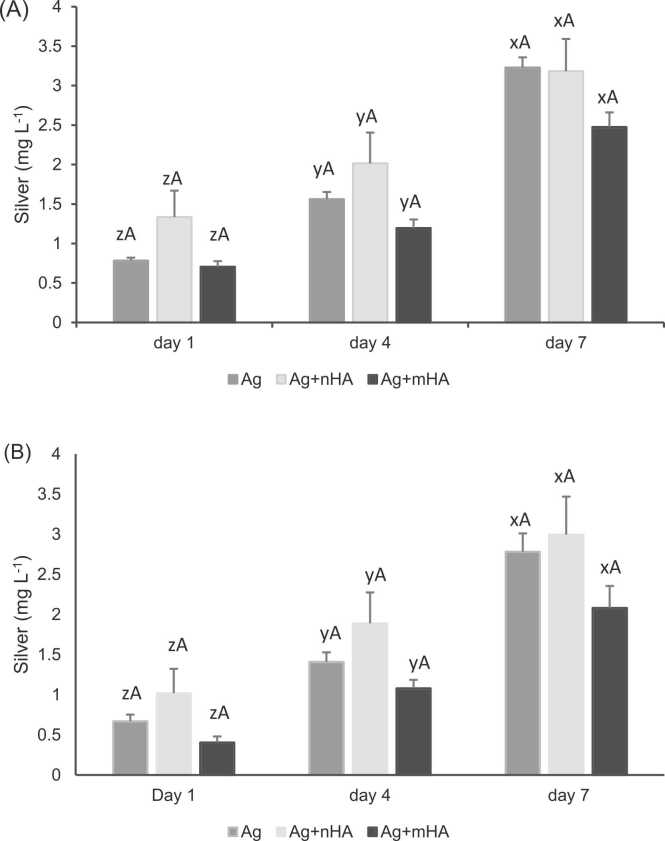
Cumulative silver release from the discs to the external media over 7 days (A) before, and (B) after exposure of the osteoblast cells. Data are mean ± S.E.M. Different capital letters within time points indicate a treatment-effect, while different small letters between the time points in the same treatment indicate a statistically significant time-effect (one way ANOVA, *p* < 0.05).

Silver release to the external media was assessed by measuring the total silver in the media using ICP-MS in the conditioned media (i.e., leachates collected from the discs) before exposure to the cells, and afterwards in the cell culture media with the cells present. The silver concentration in the media of the reference control was close to the detection limit, as expected (Table 1). All the treatments containing added silver were significantly higher than the reference control (one way ANOVA, *p* < 0.05). Silver release from all the silver plated discs to the external media (conditioned media before exposure, without the cells) ranged from 0 to 7–1.6 mg L^−1^. No significant difference was found between the groups and/or between the time points in those treatments that contained silver (Table 1), except slightly more leaching of total Ag into the conditioned media at day 7 in the Ag treatment compared to day 1. Cumulative silver release over 7 days was approximately 3 mg L^−1^ in all treatments (Fig. 2), and with no statistically significant difference was located between the treatments (one way ANOVA, *p* < 0.05).

**Table 1:** Total silver concentration in the conditioned media (mg L^−1^) before and after adding the media to the cells.

Treatment	Day 1		Day 4		Day 7	
	(Before)	(After)	(Before)	(After)	(Before)	(After)
Reference control	-	0.009 ± 0.006 ^C^	-	0.012 ± 0.004^B^	-	0.008 ± 0.005^B^
Ag	0.783 ± 0.037^zA^	0.669 ± 0.082^zA^	0.779 ± 0.071^zA^	0.738 ± 0.078^zA^	1.667 ± 0.049^yA^	1.374 ± 0.123^yA^
Ag+nHA	1.335 ± 0.334^zA^	1.017 ± 0.305^zA^	0.678 ± 0.058^zAB^	0.871 ± 0.11^zA^	1.169 ± 0.074^zA^	1.105 ± 0.153^zA^
Ag+mHA	0.705 ± 0.071^zA^	0.406 ± 0.074^yB^	0.490 ± 0.053^zB^	0.674 ± 0.048^zA^	1.282 ± 0.085^zA^	1.002 ± 0.196^zA^

Data are mean ± S.E.M. (*n* = 6). Different capital letters within the column indicate significant difference between treatments, while different small letters within the row indicate a significant difference between time points (before versus after) within treatment. One way ANOVA, *p* value < 0.05. Two way ANOVA showed that there was a time x treatment effect on silver release (*p* < 0.05). The reference control only shows “after” values as it was not pre-conditioned with silver to have “before” values (i.e., not exposed to media containing leached silver).

Silver concentration in the external media (after exposure, with cells present) was generally lower than that of the initial conditioned media, with total Ag in the cell media ranging from 0.4 to 1.4 mg L^−1^. Although, the difference in the media before and after exposure of the cells is most apparent on day 7 for the Ag treatment and Ag+mHA treatment, but not the Ag+nHA treatment (Table 1). For the Ag alone treatment, the release of Ag into the media with the cells gradually increased over 7 days and it was significantly higher at day 7 compared to day 4 and day 1 (one way ANOVA, *p* < 0.05). There was no significant difference in silver release from Ag+nHA over 7 days, however, silver release in Ag+mHA significantly increased (one way ANOVA, *p* < 0.05) at day 4 and 7 compared to day 1 (Table 1). There were no significant differences in the cumulative total Ag in the cell media (Fig. 2B). Since there was two factors affecting the silver release (treatment and time), a two-way ANOVA was performed and showed that both factors have a significant effect on silver release (*p* values < 0.05).

### 3.3 Silver accumulation, cell health and morphology after 7 days exposure

**Fig. 3:**
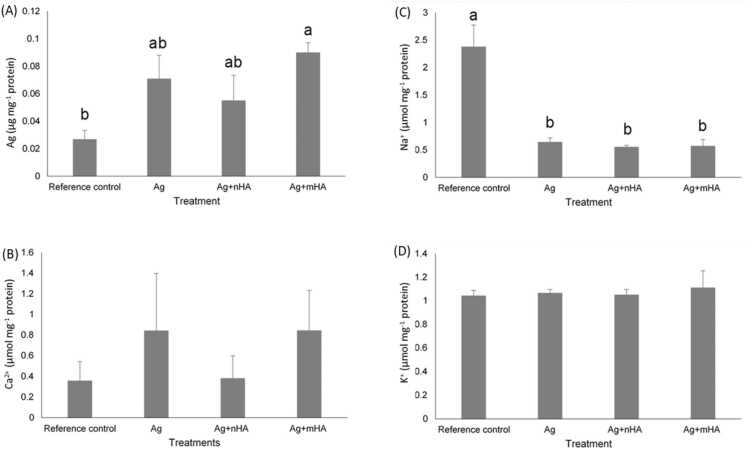
Concentration of (A) silver, (B) Ca^2+^, (C) Na^+^, and (D) K^+^, after 7 days in the cell homogenate. Data are mean ± S.E.M, (*n* = 6). Different letters indicate significant difference with each element (one way ANOVA, *p* < 0.05). Unlabelled means no significant difference between the treatments.

The Ag and electrolyte concentrations were measured in the cell homogenates (Fig. 3). The cell homogenate from the reference control (cells only, no Ag exposure) showed a trace background of total Ag, as expected (Fig. 3). However, the concentration of total Ag in the cell homogenates from all the Ag-containing treatments was modest, at around 0.1 µg mg protein^−1^ or less at day 7, indicating that only small fraction of the silver in the exposure media was accumulated by the cells (Fig. 3). Only the homogenates from the Ag+mHA treatment had statistically greater total Ag concentrations than the reference control. Compared to the unexposed reference control cells, all the Ag-containing treatments also caused some statistically significant (one way ANOVA, *p* < 0.05) depletion of the cell homogenate Na^+^ concentration, but there was no effect of the different Ag-containing treatments. There were no effects on the cell homogenate Ca^+^ or K^+^ concentrations (Fig. 3). There were no changes in the Na^+^ or K^+^ concentrations in the cell media (Table 2), but there were some transient changes in cell media Ca^2+^ concentration between days 1 and 4, with the Ag+nHA treatment notably showing some consistently lower values of total Ca^2+^ in the media compared to the reference control or Ag treatment at day 4 and 7 (Table 2).

**Table 2:** Concentration of Ca^2+,^ Na^+^ and K^+^ and enzyme activity in the external media covering the osteoblast cells over 7 days.

Element	Treatment	Day 1	Day 4	Day 7
Ca^2+^ (mmol L^−1^)	Reference control	1.15 ± 0.12^Aa^	1.83 ± 0.07^Ab^	1.72 ± 0.09^Ab^
	Ag	0.76 ± 0.18^Ba^	2.01 ± 0.07^Ab^	1.97 ± 0.15^Ab^
	Ag+nHA	0.64 ± 0.05^Ba^	1.11 ± 0.07^Bb^	0.85 ± 0.13^Bb^
	Ag+mHA	1.18 ± 0.08^Aa^	1.78 ± 0.09^Ab^	1.59 ± 0.20^Ab^
Na^+^ (mmol L^−1^)	Reference control	140.75 ± 4.17	159.81 ± 9.08	148.10 ± 2.42
	Ag	115.51 ± 13.24	146.58 ± 4.75	137.67 ± 11.30
	Ag+nHA	121.91 ± 2.30	146.39 ± 7.90	132.04 ± 14.58
	Ag+mHA	123.65 ± 2.50	160.78 ± 3.12	141.53 ± 13.86
K^+^ (mmol L^−1^)	Reference control	6.23 ± 0.14	7.25 ± 0.39	6.64 ± 0.09
	Ag	5.53 ± 0.62	7.12 ± 0.22	6.62 ± 0.52
	Ag+nHA	5.80 ± 0.10	7.09 ± 0.40	6.35 ± 0.68
	Ag+mHA	5.89 ± 0.10	7.70 ± 0.15	6.80 ± 0.66
LDH activity (nmol min^−1^ mL^−1^)	Reference control	0.343 ± 0.198	0.317 ± 0.158	0.582 ± 0.291
	Ag	0.449 ± 0.206	0.925 ± 0.383	0.899 ± 0.546
	Ag+nHA	0.476 ± 0.224	3.201 ± 2.037	0.608 ± 0.341
	Ag+mHA	0.185 ± 0.132	0.476 ± 0.312	0.793 ± 0.332
ALP activity (nmol min^−1^ mL^−1^)	Reference control	0.155 ± 0.043	0.209 ± 0.070	0.055 ± 0.026
	Ag	0.092 ± 0.023	0.093 ± 0.025	0.066 ± 0.029
	Ag+nHA	0.035 ± 0.011	0.053 ± 0.008	0.007 ± 0.003
	Ag+mHA	0.064 ± 0.021	0.059 ± 0.020	0.006 ± 0.001

Data are mean ± S.E.M (*n* = 6). Different capitals letters within the column indicate statistically significant difference from each other, while different small letters within the raw indicate a significant difference (one way ANOVA *p* <0.05 for the electrolytes). Unlabelled means no significant difference. Two-way ANOVA showed that there was a significant time and treatment effect on Ca^2+^ concentration. There were no treatment or time effects in the enzyme activities (Kruskal Wallis test, *p* > 0.05), The detection limit for LDH and ALP activities were 0.549 and 0.004 nmol min^−1^ mL^−1^ respectively.

**Fig. 4:**
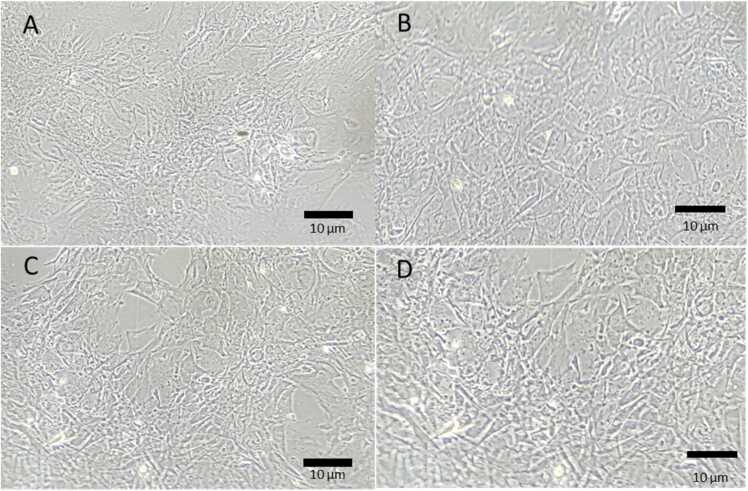
Microscopical images of the osteoblast cells after 7 days exposure to silver. (A) reference control, (B) Ag, (C) Ag+nHA, (D) Ag+mHA. Note the cells are healthy with no evidence of cell swelling or membrane.

**Fig. 5:**
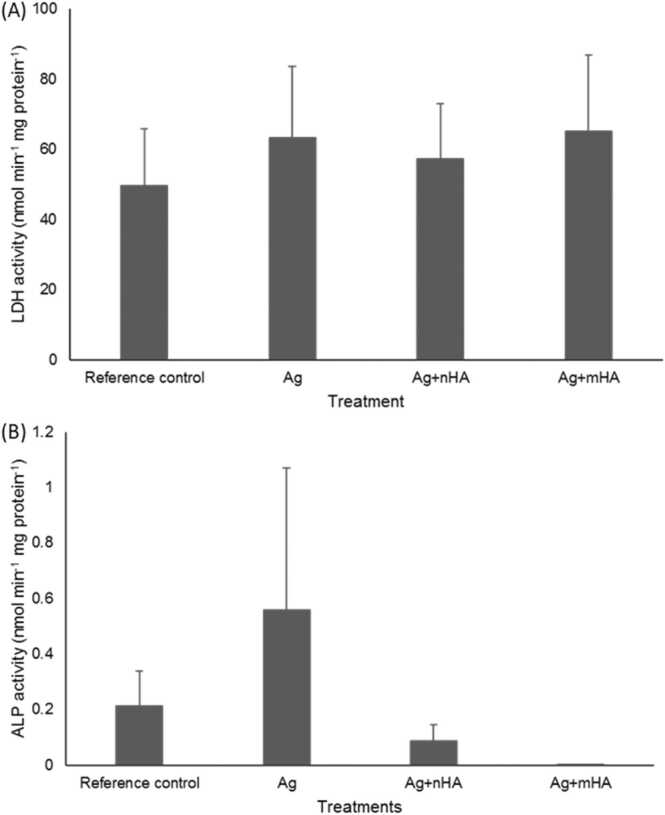
Cell homogenate enzyme activities at day 7. (A) Lactate Dehydrogenase, (B) Alkaline phosphatase. Data are mean ± S.E.M., (*n* = 6). There were no significant differences between the treatments (Kruskal Wallis test, *p* > 0.05).

The cells were also examined under the light microscope after 7 days (Fig. 4). The osteoblasts were confluent and appeared healthy in all treatments, with no obvious signs of membrane damage or cell swelling. In addition, there was no significant difference (one way ANOVA, *p* > 0.05) in protein content of the cell homogenates between the treatments, with protein concentrations of 0.14 ± 0.01, 0.12 ± 0.00, 0.12 ± 0.01, and 0.13 ± 0.01 mg mL^−1^ for the reference control, Ag, Ag+nHA, and Ag+mHA treatments respectively. Although the silver exposure was confirmed in the media, there was not an appreciable leak of LDH or ALP activity into the external media compared to the reference control (Table 2). Similarly, there was no significant differences (Kruskal Wallis, *p* > 0.05) in the LDH activity in the cell homogenate between the reference control and the other treatments (Fig. 5). As expected for immature cells, a small background of ALP activity was detected with values being slightly higher than the detection limit in the cell homogenates, but no treatment effect was observed (Fig. 5B).

## 4 Discussion

In this study, the potential toxicity of dissolved silver in conditioned media derived from silver-coated titanium discs was explored. The experimental approach exposed human primary osteoblast cells to the conditioned media so that any direct toxicity of silver in the media could be identified, and differentiated from our previous study where contact toxicity was possible with the cells grown directly onto the silver-coated titanium alloy discs [11]. Overall, while some total Ag was released by dissolution into the conditioned media, it was not toxic to the osteoblasts suggesting that the media remained biocompatible with these human cells.

### 4.1 Silver release to the conditioned media

The coatings used in this study were designed to provide a slow release of Ag into the surrounding media in order to impart biocidal properties to microbes [9], but also at a lower enough total Ag concentration to avoid toxicity to osteoblasts or other human cells [11]. In the present study, between 0 and 7–1.6 mg L^−1^ of total Ag was released into the conditioned media (Table 1), broadly in keeping with our previous observations with DMEM over 7 days [1–4 mg L^−1^ depending on treatment with cells present [11]]. Dialysis studies in DMEM (dialysis tubing pore size < 2 nm) showed that only a tiny fraction (about 3 µg L^−1^ or less over 24 h) was truly dissolved Ag [11], in keeping with these calculations. Indeed, Ag salts instantaneously form insoluble AgCl particles in high ionic strength media [12]. From the measured total Ag in the conditioned media and speciation calculation above, the osteoblasts would be presented with between 7 and 16 µg L^−1^ as the Ag^+^ ion. The standard DMEM recipe used here also contained amino acids, including 2 mmol L^−1^ histidine, which avidly binds Ag^+^ [27], as would the –SH residues in the 10 % FBS supplemented to the media. Consequently, there would be negligible free Ag^+^ in the conditioned media.

There were also some effects of the different surface coatings on the Ca^2+^ concentration in the conditioned media, which showed a significant reduction in the Ag+nHA treatment compared to others by day 7 (Table 2). This is best explained by adsorption of Ca^2+^ onto the surface of the HA leading to the formation of Ca(OH)_2_. This phenomenon is well-known and previously reported for HA-containing materials [9], [28] and in our previous work with DMEM with osteoblasts [11].

### 4.2 Absence of silver toxicity to osteoblasts from conditioned media

The absence of silver toxicity to the osteoblasts was confirmed by the normal morphology and growth of the cells to confluence (Fig. 4), and no appreciable leak of LDH or ALP activity in the external media compared to controls (Table 2). Furthermore, there were no effects on homogenate enzyme activities (Fig. 5) which are consistent with our previous report where the cells were grown directly on the same materials [11]. ALP activity was low in the cell homogenate and this is expected for immature osteoblasts. For example, [29] also observed that the ALP activity in the osteoblast cell homogenate after 3 days culture was less than 1 nmol min^−1^ mg protein-^1^. There was only limited effects of the Ag-containing treatments on homogenate electrolyte concentrations (Fig. 3). For the latter, there was a small, but statistically significant, depletion of homogenate Na^+^ concentration (Fig. 3C). Dissolved Ag is known to inhibit the Na^+^/K^+^-ATPase in the cell membrane [23], and this would prevent the normal active efflux of Na^+^, leading to elevations of intracellular Na^+^ (Hussain *et al*., 1994). However, this was not observed, and instead the Na^+^ depletion might be explained by Ag-dependant block of the passive Na^+^ influx through the epithelial sodium channel [30], which is also present in osteoblasts [31]. In addition, the soluble free silver ions were not enough to inhibit the Na-K transporter given the presence of chloride and proteins with sulphur ligands. Nonetheless, given the normal cell morphology (Fig. 4), this small decrease in homogenate Na^+^ was well within the osmoregulatory capacity of the cells.

The cell health was good despite the presence of measurable amounts of total Ag in the culture showing that the cells were exposed (Table 1). This is best explained by low bioavailability of the total Ag in the media to the osteoblasts. Indeed, the homogenate Ag concentrations remained low (about 0.1 µg mg^−1^ protein or less, Fig. 3A), with only the Ag+mHA treatment being different to the reference control. This is also in keeping with the silver speciation arguments above. Interestingly, [32] exposed osteoblasts to supernatants from a silver nanoparticle (Ag NP, size 50 nm) suspension in minimal essential alpha medium (α-MEM) supplemented with 10 % FBS plus 1 % antibiotics. The supernatants, prepared by centrifugation, were assumed to be free of the original particles and therefore to contain only dissolved Ag released by dissolution from the particles. They found that the lC_50_ value for osteoblast viability after 72 hours exposure to the supernatants in culture media was 0.068 mmol L^−1^ (7.34 mg L^−1^) of total Ag. If 10 % of the IC_50_ is regarded as sublethal, then the sublethal concentration of total Ag for osteoblasts in cell culture media is around 1.46 mg L^−1^, within the exposure reported here (Table 1). Crucially, this implies that the 20–30 % reduction in cell viability we observed from osteoblasts growing *in situ* on the same Ag-coated titanium alloys [11] was not caused by dissolution of Ag into the media, but instead, must be associated with the preference of the cells for the roughness, crystallinity and chemical composition of the implant surface. Yet the materials are shown to be biocidal, preventing the growth of *Streptococcus sanguinis* [9].

### 4.3 Conclusions and clinical perspective

The clinical safety concern that the deliberate slow release of a biocidal amount total Ag from Ag-containing coatings on medical grade titanium alloy may be hazardous to human cells or be deleterious to the biocompatibility seems unfounded in this specific case and conditions. The silver in the conditioned media had limited bioavailability and no appreciable toxicity to the osteoblasts. This observation will hopefully give some confidence that biocidal properties of the implant material are not contradictory to the role of osteoblasts in the osseointegration and subsequent wound healing after dental implant placement surgery. While further refinements of the Ag+nHA material may improve osteoblast proliferation further, the next step will be to test the materials *in vivo* in an animal model.

## CRediT authorship contribution statement

**Ranj Salaie:** Writing – original draft, Methodology, Data curation. **Richard D. Handy:** Writing – review & editing, Project administration, Funding acquisition. **Christopher Tredwin:** Project administration. **Alexandros Besinis:** Methodology.

## Declaration of Competing Interest

The authors declare no conflict of interest

## Data Availability

No data was used for the research described in the article.
