# Supplementary material for: Low toxicity of dissolved silver from silver-coated titanium dental implants to human primary osteoblast cells
Source: Toxicol Rep. 2024 Oct 18;13:101776. doi: 10.1016/j.toxrep.2024.101776 (PMC11532920; doi:10.1016/j.toxrep.2024.101776)
Supplement: Supplementary file 1 — Supplementary material [file mmc1.docx]

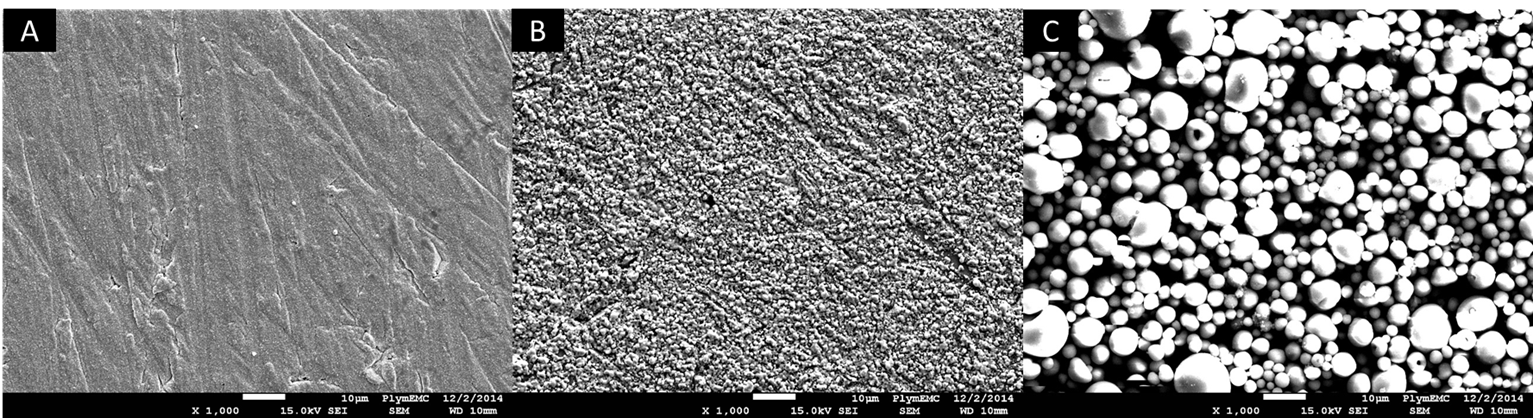


Figure S1: SEM images of coated titanium discs; (A) silver plated titanium alloy disc surface. (B) Ag+nHA disc surface. Note the nHA particles have successfully produced an even coating on the silver plated titanium. The mHA particles have successfully been coated on the substrate.


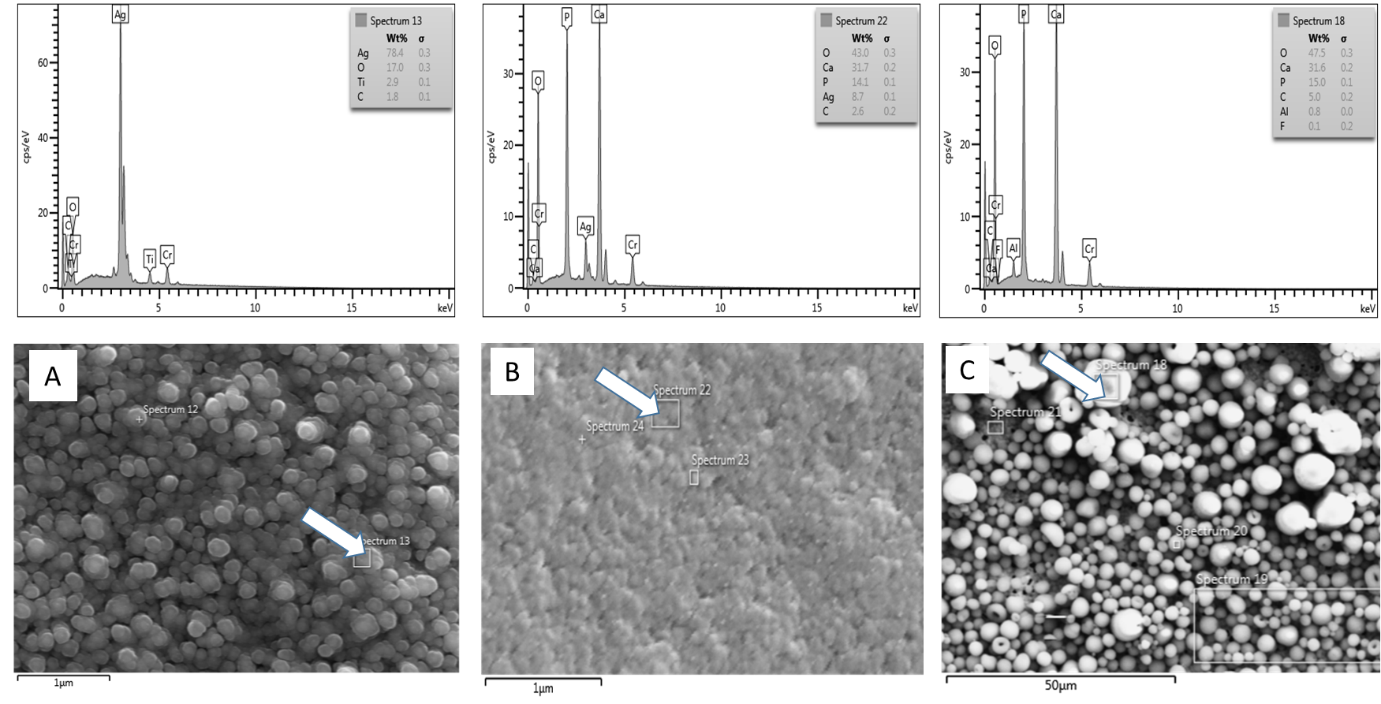


Figure S2: EDS images of coated titanium discs; (A) silver plated titanium (Ag), (B) silver plated plus nano HA (Ag+nHA), (C) silver plated plus micro HA (Ag+mHA).
